# Significance of ectopic intrathyroidal thymic tissue detected on ultrasound in different paediatric age groups: a proposed classification to guide investigation and management

**DOI:** 10.1007/s00431-025-06121-9

**Published:** 2025-04-08

**Authors:** Hannah Jeffery, Karen D. Bosch, Caroline Brain, Tom Kurzawinski, Tim Beale, Xin-Ying Kowa, Tarek Abdel Aziz

**Affiliations:** 1 Department of Endocrine Surgery, University College London Hospitals NHS Foundation Trust, 250 Euston Road, London, NW1 2PG UK; 2 Department of Paediatric Endocrinology, Great Ormond Street Hospital, London, UK; 3 Department of Radiology, University College London Hospitals NHS Foundation Trust, London, UK

**Keywords:** Thyroid, Thymus, Thyroid cancer

## Abstract

Ectopic aberrant thymic tissue is most commonly found in the thyroid gland and is increasingly found incidentally due to the widespread use of ultrasound. Correct identification of this benign pathology on ultrasound can avoid the morbidity associated with fine needle aspiration (FNA) and anxiety associated with a possible cancer diagnosis. A case series of 21 children found to have likely intrathyroidal thymic tissue on ultrasound scan of the neck between 2013 and 2024 at the University College London Hospital. Twenty-one children aged between six months and ten years old, 52% male and 48% female. The scans were performed following referral for a neck lump or cervical lymphadenopathy (N = 15), sore throat/cough (N = 4), pre-thyroidectomy scan for a Multiple Endocrine Neoplasia 2 patient (N = 1), and post cystic hygroma excision routine scan (N = 1). These well-defined hypoechoic foci ranged in size from 3 to 14 mm and direct comparison to normal thymic tissue was possible in 19/21 (90%). Three patients (14%) went on to have FNA to rule out papillary thyroid cancer (PTC); other cases had interval scans at median six months to confirm stable appearances. *Conclusion*: All patients in our study with intrathyroidal ectopic thymic tissue were younger than 10 years. We suggest a tailored management approach based on the age at presentation, presence of clear thymic tissue for comparison and past/family history. Given that it is unlikely for such tissue to be present in older children, an FNA would be recommended to exclude underlying thyroid cancer in children over 14 years.

**Table:** 

**What is known:** **• ***Intrathyroidal thymic tissue is a common variant that will be increasingly incidentally picked up on ultrasound scans in chlidren.*
**What is new:** **• ***We suggest separation of children with ectopic intrathyroidal thymic tissue into three groups based on age and the ability to compare directly with normal thymic tissue.*

**What is known:**

**• ***Intrathyroidal thymic tissue is a common variant that will be increasingly incidentally picked up on ultrasound scans in chlidren.*

**What is new:**

**• ***We suggest separation of children with ectopic intrathyroidal thymic tissue into three groups based on age and the ability to compare directly with normal thymic tissue.*

## Introduction

The thymus is a bilobed organ located within the anterior mediastinum. Its main function is immune regulation as the primary site of T-lymphocyte development, and it also has a proposed endocrine role. Embryologically, the lobes of the thymus originate from the third pair of pharyngeal pouches in the pharyngeal region of the embryo migrating caudally and medially to their final location within the thorax and fusing in the midline [1]. Due to the proximity of the thyroid diverticulum from which the thyroid forms, and the third pharyngeal pouches, the descents of the thymus and the thyroid are closely related [2]. Thymic tissue can remain anywhere along its path of descent, with the prevalence of intrathyroidal thymic tissue in children estimated at 1%–2.6% [3, 4]. Like the thymus, intrathyroidal tissue also undergoes involution with age.

Ultrasonography is a safe and non-invasive form of neck imaging and is first line in the investigation of neck lumps including thyroid nodules. Children commonly present with enlarged cervical lymph nodes, and the increasing availability and use of ultrasound has resulted in increasing incidental findings of thyroid nodules [5]. Thyroid nodules remain relatively uncommon in children, but, when identified, they have a higher risk of malignancy (22–26%) than in adults (5–10%) [6].

Papillary thyroid cancer (PTC), the commonest type of thyroid cancer in children accounting for 90% of cases [6], has similar sonographic features to intrathyroidal thymic tissue. Both appear hypoechoic compared to the strap muscles, and ectopic thymic tissue contains scattered echogenic foci which can mimic the microcalcifications often seen in PTC. However, unlike intrathyroidal thymic tissue, PTC is unlikely to shrink with age; it may remain the same size for some time, enlarge and/or metastasize to cervical lymph nodes. When ectopic thymus is mistaken for a potentially malignant lesion or diagnostic uncertainty remains, patients undergo fine needle aspiration (FNA), often under general anaesthetic.

The purpose of this study is to evaluate the diagnosis, investigation and management of patients with intrathyroidal ectopic thymic tissue referred to a tertiary centre over a 10-year period, raise awareness of this diagnosis, ascertain whether it is safe to monitor low-risk patients rather than confirming the diagnosis with FNA.

## Methods

A retrospective search of the electronic patient data platform EPIC and the radiology information system Soliton at a tertiary paediatric referral centre between 2013 and 2022 was performed. This included ultrasound scans of patients aged less than 16 years at the time of the scan with text in the report including “thymus” or “thymic”.

Cases of suspected or confirmed intrathyroidal thymic tissue were included in the analysis. Data were collected using EPIC on patient demographics, reason for referral, and follow up, including whether the patient subsequently underwent FNA to rule out PTC. The decision to perform FNA was a clinical decision and not based on set criteria.

Microsoft Excel was used to collect the data, the data were anonymised, and analysed using descriptive statistics.

The research was conducted ethically in accordance with the World Health Association Declaration of Helsinki.

## Results

Over the ten-year period, a total of 21 cases were identified. All children were under the age of 10 years. Demographics of these patients are shown in Table 1.

**Table 1:** Patient demographics

Demographic		Number (%)
Age at referral	< 12 months	1 (5%)
	1–3 years	2 (10%)
	3–10 years	18 (85%)
	> 10 years	0 (0%)
Sex	Male	11 (52%)
	Female	10 (48%)
Ethnicity	Asian	8 (38%)
	White	7 (33%)
	Mixed	4 (19%)
	Not recorded	2 (10%)
Presentation	Neck lump	7 (33%)
	Cervical lymph nodes	8 (38%)
	Cough/sore throat and others	4 (19%)
	Asymptomatic	2 (10%)

The most common reason for referral was following external ultrasound that identified a thyroid nodule (12 cases). Although these patients were referred to our centre for FNA to exclude a thyroid malignancy, in many cases FNA was not considered necessary following further ultrasound assessment. In general, a palpable neck lump was the commonest presentation, which was either a thyroid nodule in 7 cases (33%) or an enlarged cervical lymph node in 8 cases (38%). In those patients with enlarged cervical lymph nodes, the intrathyroidal thymic tissue was an incidental finding on ultrasound. One patient with multiple endocrine neoplasia type 2 (MEN2) was found to have intrathyroidal thymic tissue on pre-thyroidectomy ultrasound scan (Table 1).

Ectopic thymus tissue was equally likely to be found in the left (10 cases, 48%) or right (11 cases, 52%) thyroid lobes. Where further descriptors of location were provided in the report (11 cases), ectopic tissue was most commonly found posteriorly in the inferior thyroid pole. The maximum diameter of the lesions ranged in size from 3 to 14 mm (median 6 mm, IQR 4.75 mm).

**Fig. 1:**
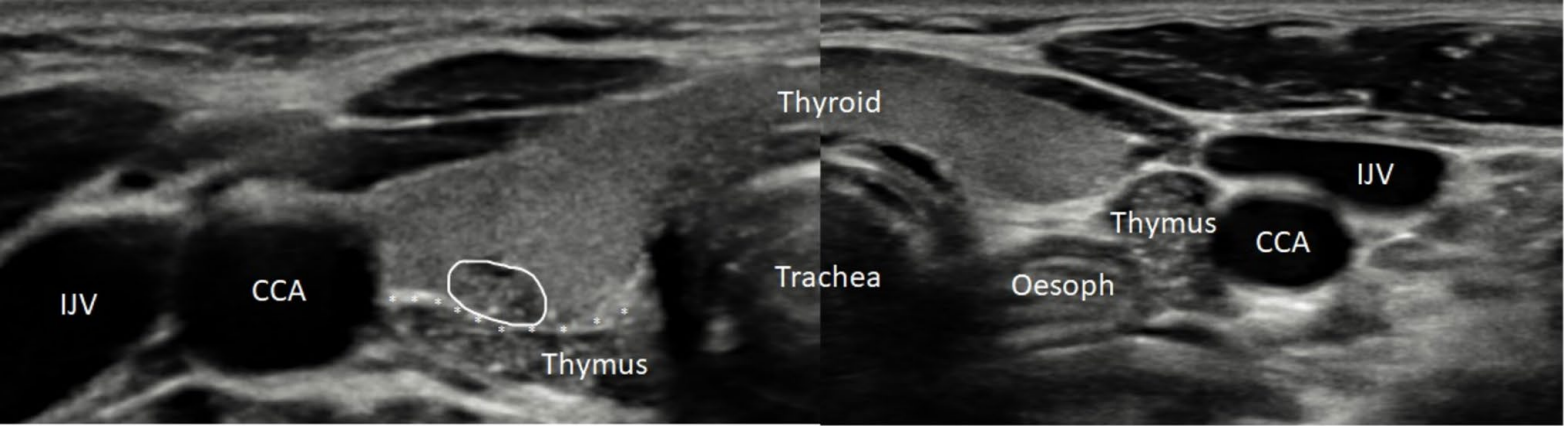
Transverse ultrasound of a 3-year-old patient. Right-sided subcapsular intra-thyroid thymic remnant (solid outline) abuts the posterior capsule of the thyroid gland. The intra-thyroid thymic remnant demonstrates the same sonographic appearance as native thymus present within the lower neck which is visualised posterior to this and separated by an intervening tissue plane (asterisks). Native thymus is also present within the left infra-thyroid location. Thymic tissue in the paediatric age group is hypoechoic with internal echogenic tissue and flecks (‘starry sky’ appearance). CCA = common carotid artery. IJV = internal jugular vein. Oesoph = oesophagus

**Fig. 2:**
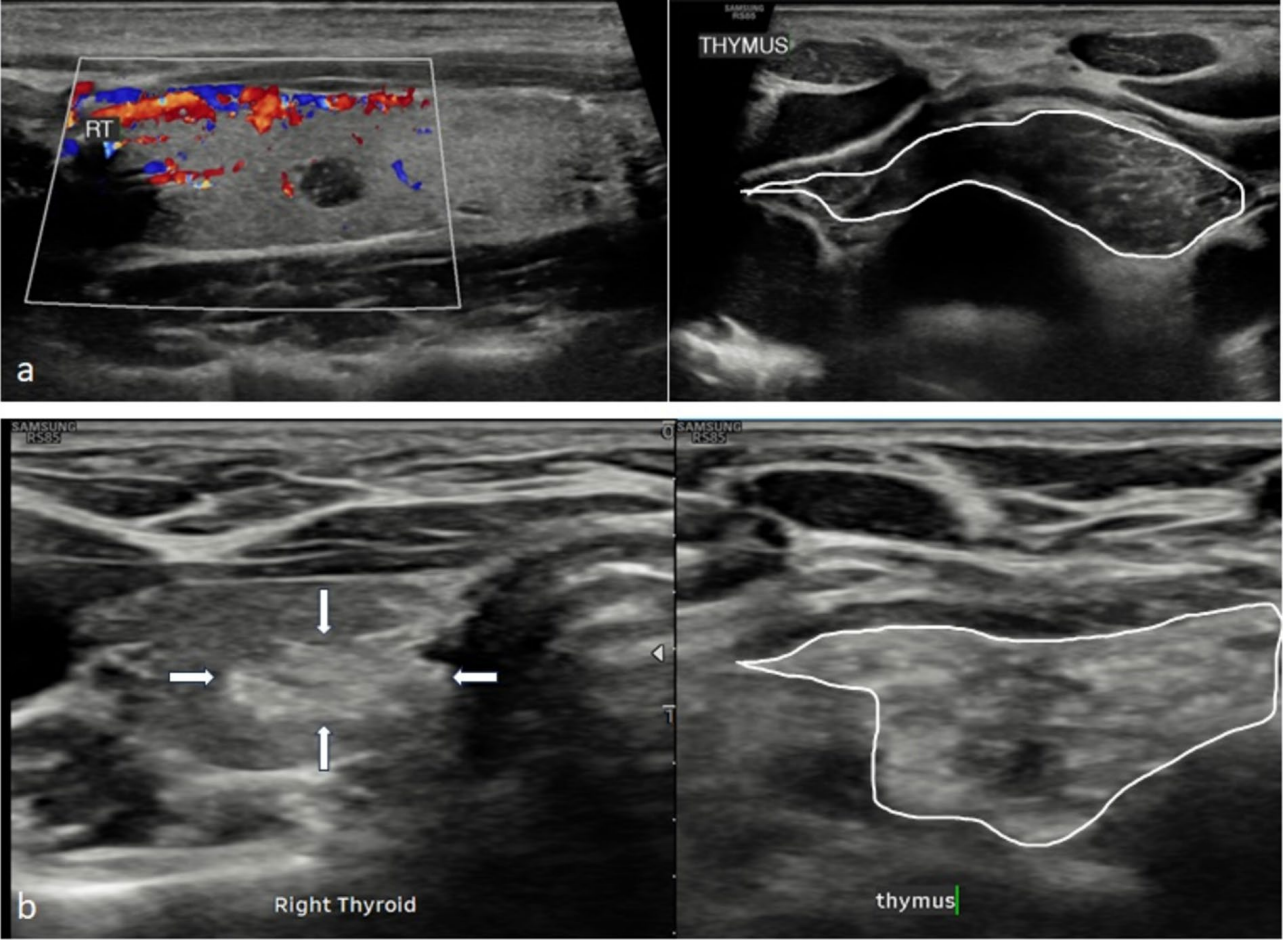
Spectrum of appearances of intra-thyroid thymic remnant juxtaposed against native thymus. (**a**) Ultrasound of a 12-year-old patient. Longitudinal image of the right thyroid lobe with an avascular, predominantly hypoechoic intra-thyroid thymus (within the colour box) that contains only a couple of echogenic flecks. The adjacent transverse image depicts the native thymus (solid outline) within the superior mediastinum which exhibits the same sonographic appearance. (**b**) Ultrasound of a 6-year-old patient. The transverse image depicts an almost uniformly echogenic right-sided intra-thyroid thymus (thick white arrows) and native predominantly echogenic thymic tissue (solid outline) within the superior mediastinum

**Fig. 3:**
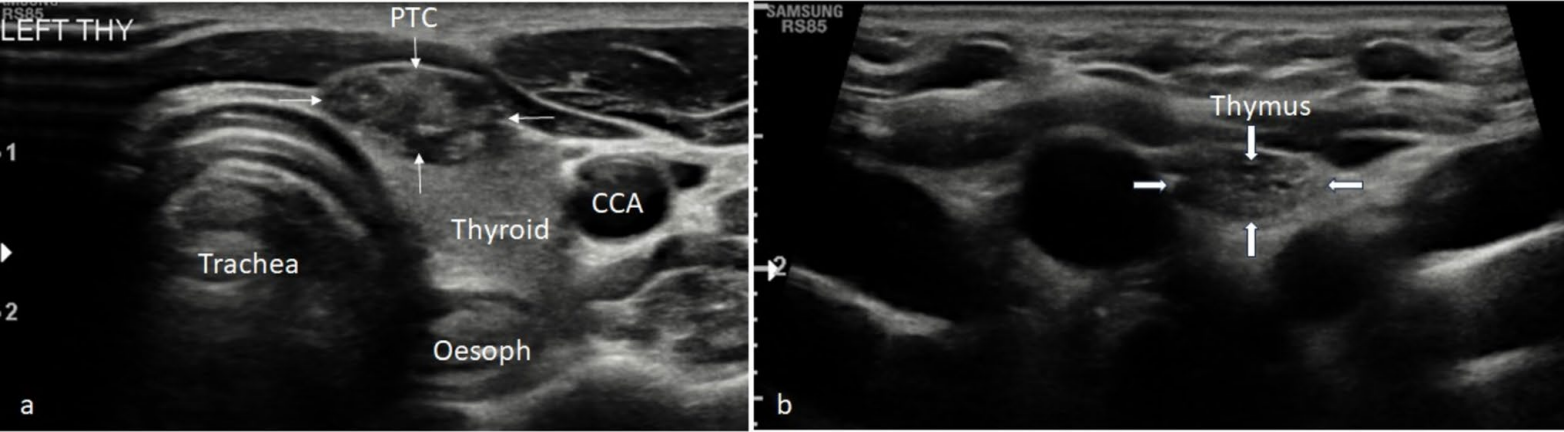
Transverse ultrasound of a 17-year-old patient. (**a**) A left-sided papillary tumour (thin white arrows) demonstrates mass effect elevating the anterior capsule of the thyroid lobe. The tumour is heterogeneous with a mixed hypoechoic-echogenic appearance and internal hyperechoic flecks in keeping with non-shadowing microcalcifications (a BTA U5 feature). (**b**) Involuting thymus (thick white arrows) within the superior mediastinum is relatively hypoechoic with no residual echogenic foci reflecting its increased fat composition. This is visibly different in appearance compared to the tumour. CCA = common carotid artery. Oesoph = oesophagus. PTC = papillary thyroid tumour

Of the 21 patients, 16 of the nodules were reported as ‘hypoechoic’, ‘hyporeflective’ or ‘low reflectivity’; and 19 scans demonstrated ‘echogenic’, ‘hyperechoic’ or ‘hyperreflective’ foci or echoes. Direct comparisons with the sonographic appearance of the patient’s thymus were possible in 19 (90%) cases, either on initial or follow-up ultrasound, to aid diagnosis (Table 2). Examples of ultrasound appearance of intrathymic thyroid tissue are shown in Figs. 1 and 2, and these can be compared with appearances of PTC in a child shown in Fig. 3.

**Table 2:** Ultrasound characteristics and follow-up of each patient

Patient	Ultrasound characteristic				Follow-up			
	**Shape**	**Echogenicity**	**Hyperechoic foci**	**Direct comparison to patients own thymus**	**FNA**	**Duration of follow-up (months)**	**Follow-up scans**	**Follow-up US appearance**
1	Linear area	Hypoechoic	Present	Present		22	1	Stable
2	Geographically shaped	Hypoechoic	Present	Present		Ongoing (MEN2 A)	0	-
3	Ovoid focus	Hypoechoic	Present	Present		3	2	Stable
4	Ovoid	Echogenic	Present	Present		Ongoing (Sjogren’s)	0	-
5	Ovoid	Hypoechoic	Present	Present		6	0	-
6	Well defined	Low echogenicity	Present	Present		39	5	Stable
7	Ovoid	Echogenic	Present	Present		6	0	-
8	Linear	Hyporeflective	Present	Present		7	1	Decreased in size
9	Oval shaped	Echogenic	Present	Present	Yes	57	5	Stable
10	Elongated region	Hypoechoic	Present	Present		0	0	-
11	Oval	Hyporeflective	Absent	Absent		15	2	Stable
12	Oval	Hypoechoic	Present	Present		0 (DNA 6-month scan)	0	-
13	Slightly irregular	Hypoechoic	Present	Present	Yes	12	2	Stable
14	Ovoid	Hypoechoic	Present	Present		0	0	-
15	Ovoid	Hyporeflective	Present Apr 17, Absent Oct 17	Present		6	2	Decreased in size
16	Ovoid	Hypoechoic	Present	Present		0	0	-
17	Ovoid	Echogenic	Present	Present		0	0	-
18	Bilobed, well defined nodule	Hyporeflective	Present	Absent		0	0	-
19	Ovoid	Low reflectivity	Present	Present		1	1	Disappeared
20	Ovoid	Hypoechoic	Present	Present	Yes	0	0	-
21	Geographic	Hypoechoic	Present	Present	No	4	1	Stable

One patient had a second larger nodule within the same lobe of the thyroid described as a ‘well defined, slightly hypoechoic vascular nodule’ of indeterminate significance. Another patient had a left paratracheal nodule inferior to the left lobe of the thyroid, containing similar echogenic foci to the thyroid nodule, likely to represent another small focus of ectopic thymic tissue. 12 patients had benign reactive lymphadenopathy on ultrasound. One patient had diffusely abnormal salivary glands later diagnosed as Sjogren’s disease on biopsy.

Three patients (14%) went on to have FNA to rule out PTC. A 7-year-old male patient with 2 nodules in the left lobe underwent FNA in October 2013. Biopsies were taken from both the 8-mm vascular nodule and the 6-mm nodule containing echogenic foci. The former was a benign thyroid nodule (Thy2) on cytology; the latter consisted of blood and aggregates of lymphoid cells, mainly small and medium sized, with occasional specks of calcium. Immunohistochemistry was performed and the majority of lymphoid cells stained positively with CD3, confirming that the infiltrate was consistent with thymic origin.

The second patient to undergo FNA in May 2014 was a 5-year-old female with a 6-mm nodule within the posterior aspect of the mid right lobe of the thyroid. Cytology comprised lymphocytes and germinal centre fragments, with no evidence of malignancy. Immunohistochemistry was performed to try and distinguish whether the lymphocytes present were part of a lymph node or whether they could be of thymic origin; however, there was insufficient material left for adequate interpretation. The patient subsequently went on to have five annual follow-up scans which all showed unchanged appearances of the nodule, prior to discharge back to local hospital.

The third patient underwent FNA in January 2017. This 5-year-old boy had an initial ultrasound scan showing a diffusely hypoechoic nodule measuring 4 × 5 mm containing multiple hyperechoic foci, which may be in keeping with microcalcification. Cytology of the sample showed predominantly lymphocytes at various stages of maturation, with occasional germinal centre fragments and very rare sheets of epithelial cells showing no cytological atypia. There were no features to suggest PTC. The patient had follow-up scans at three- and ten-months post biopsy which showed unchanged appearances of the nodule.

Of the 18 (86%) patients who did not undergo FNA, 14 patients either underwent interval scan at our centre or were recommended follow up scan be conducted at six months at their local hospital. Ten patients had interval scans at our centre within 1 and 22 months of the initial scan (median time to first follow up scan 6 months). All interval scans demonstrated either stable appearances of the nodule (seven patients), a reduction in size (two patients), or disappearance of the nodule (one patient). One patient underwent a prophylactic thyroidectomy as planned for MEN2; the histology showed C-cell hyperplasia, with no evidence of medullary carcinoma or micro-carcinoma.

## Discussion

The incidental finding of ectopic intrathyroidal thymic tissue in children is becoming increasingly common due to the widespread use of ultrasonography and this incidence is only expected to rise in the future. The clinical concern is that this finding is often misinterpreted as a thyroid tumour with a differential diagnosis of thyroid cancer and is communicated as such to the child and parents during the initial investigation which is usually performed by a non-specialist head and neck radiologist.

There is a huge disparity in management of this condition ranging from unindicated surgery resulting in medicolegal negligence claims to unnecessary long term follow up and/or FNA biopsies. There are no current guidelines or classification systems available to help manage this condition as most evidence is based on expert opinion. A better understanding of the natural course of both thymic gland development and involution in children and teenagers coupled with an equally good understanding of the risk of thyroid cancer in children is warranted. This knowledge would allow an age-dependent selective approach tailored to the individual patient avoiding unnecessary anxiety, overtreatment and/or intervention.

The UK National Clinical Consensus Guideline for paediatric differentiated thyroid carcinoma recommends “undertaking ultrasound-guided FNA on a thyroid nodule reported on ultrasound as U3 — indeterminate, U4 — suspicious or U5 — malignant” [6]. Although FNA is considered a safe and minimally invasive procedure, it carries a small risk of haematoma, and often requires a general anaesthetic to accurately target the lesion, and its associated risks, in the paediatric population.

We studied 21 children who were diagnosed with incidental ectopic intrathyroidal thymic tissue. The majority of patients were referred from other centres to our unit to perform an FNA biopsy to rule out a thyroid cancer. This has been the information communicated to the patient and the parents at the time due to lack of knowledge of a possible benign intrathyroidal thymic entity.

Accurate anatomical imaging by highly skilled head and neck radiologists is key in making a thorough assessment but other individual patient factors also need to be considered. Most important of these are patient’s age, personal and family history and access to previous ultrasound scans for comparison which is sometimes a logistical issue with distant referrals. The age of the child at presentation would be the most important determinant in our opinion. However, the upper age limit of children is often unclear but of significance in developmental endocrinology, as thymic gland development and involution are age-related. The UNISEF and the European union define children as persons under the age of 18 years while the American academy of paediatrics has gone a step further recommending an upper limit of 21 years for the paediatric population [7, 8]. Many clinicians would agree that the upper age limit should be at puberty. This would correspond well with endocrine pathophysiology and in particular, thymic involution. This inter-paediatric separation is not well-recognised by most hospital systems who would manage individuals under 18 years as one entity being admitted to the same wards, having access to same imaging services and treatment. It is our opinion and especially for thyroid cancer/thymus evaluation that pre-pubertal children should be separated from older teenagers and young adults.

Thyroid nodules in younger children are not common. The paediatric thyroid gland would not have gone through the chronic, low grade and intermittent TSH stimulation leading to hyperplasia and with time thyroid nodularity that is in part hormonally dependent, a feature lacking in pre-pubertal children. The medical concern here would be an underlying thyroid malignancy, most commonly PTC. It is also helpful to understand the difference in thyroid cancer incidence between younger children, especially the group where ectopic intrathyroidal thymic tissue is most common – in our series under 8 years with no one diagnosed over 10 years — and those who are older; teenagers and/or young adolescents who would still be classified as children by many societies. The UK cancer statistics for children, teenagers and young adults have reported that of 128 children diagnosed with thyroid cancer in 2021, only 13 (10%) were under the age of 14 years and only 3% were underage ten [9].

Most studies in the literature have not included details on children’s ages at the time of diagnosis but rather report on a mean age. None of the children > 10 years old or teenagers who were scanned at our unit had a diagnosis of intrathyroidal thymic tissue (420 ultrasound scans annually). Other studies have similarly shown that intrathyroidal thymic tissue is rare in older children; of the case series included [2, 5, 10–21] eight include children > 10 years old, and only one included children > 14 years old. The Fukushima Health Management Survey [3] found ectopic intrathyroidal thymus in 0.6% of participants aged 10–14 years and only 0.2% of participants aged 15–19 years, compared with 1.7% and 1.8% of those aged 0–4 years and 5–9 years respectively. Similar to our study, MH Bang et al. [10] reported on 16 children with intrathyroidal thymic tissue with none of the patients aged 10 or above.

The majority of reports of intrathyroidal thymic tissue in the literature are single case studies. Of the case series reported [2, 5, 10–21], the numbers of patients ranged from 2 to 42. These are low numbers and historically intrathyroidal thymic tissue has been considered rare. However, paediatric autopsy data [4] found thymic tissue in 9 (2.6%) of 350 thyroid glands, unrelated to cause of death of the child. Furthermore, the Fukushima Health Management Survey [3] found intrathyroidal thymic tissue in 375 patients with an incidence rate of 0.99%. This suggests that intrathyroidal thymic tissue is a common variant that will be increasingly incidentally picked up on ultrasound scan.

Alongside direct comparison with the thymus, there are sonographic features that may aid differentiation of ectopic thymus from PTC. Thymic tissue appears more hypoechoic than PTC; the echogenic foci in thymic tissue are brighter than those that represent microcalcifications in PTC; and intra-nodular blood flow on Doppler is less prominent in thymic tissue than in PTC [7]. This data suggests that the diagnosis can be reliably made in low-risk cases on the basis of sonographic features alone due to feasibility of comparison of the lesion to the patient’s own thymus which occurred in 90% of cases, with only three patients requiring an FNA.

Of the 12 patients who had follow up scans at our centre, all scans showed stable or improved appearances of the nodule: six were unchanged in size, three had slightly reduced in size, one was not mentioned in the report, and one demonstrated a slight increase in size. An eventual reduction in size is to be expected, in keeping with natural involution of the thymus. This is supported by Fukushima data [3] in which prevalence was inversely proportionate with age. Small increases in size are not necessarily cause for concern: the process of involution does not begin until puberty [22] so it would be logical to expect ectopic tissue to follow the same growth pattern.

There was one case of intrathyroidal thymic tissue which was detected on an ultrasound scan performed to assess presence of medullary thyroid cancer in a patient with MEN2 A prior to a prophylactic thyroidectomy. Evidence of intrathyroidal suspicious pathology in these patients would change the surgical strategy from a prophylactic to a therapeutic approach, unnecessarily escalating the extent of surgery to include a prophylactic level 6 lymph node dissection. Our group had previously shown that such an operation is correlated with a higher risk of postoperative hypocalcaemia [23]. The recognition of such an entity may avoid such complications in this group of patients, especially given that a prophylactic thyroidectomy would usually be recommended and performed in children under 5 years and occasionally under 10 years old.

There was significant variability in the time to the first follow up scan (1–22 months), duration of follow-up (1–39 months, median follow-up 6 months), and number of follow up scans (maximum 5). The first follow-up scan was generally recommended in either three- or six-months’ time. In three patients, no follow-up was recommended. This presumably reflects a confidence in benign appearances. There is no general consensus agreement on the frequency of the interval ultrasound scans required and/or for the duration these children would need to be monitored.

**Fig. 4:**
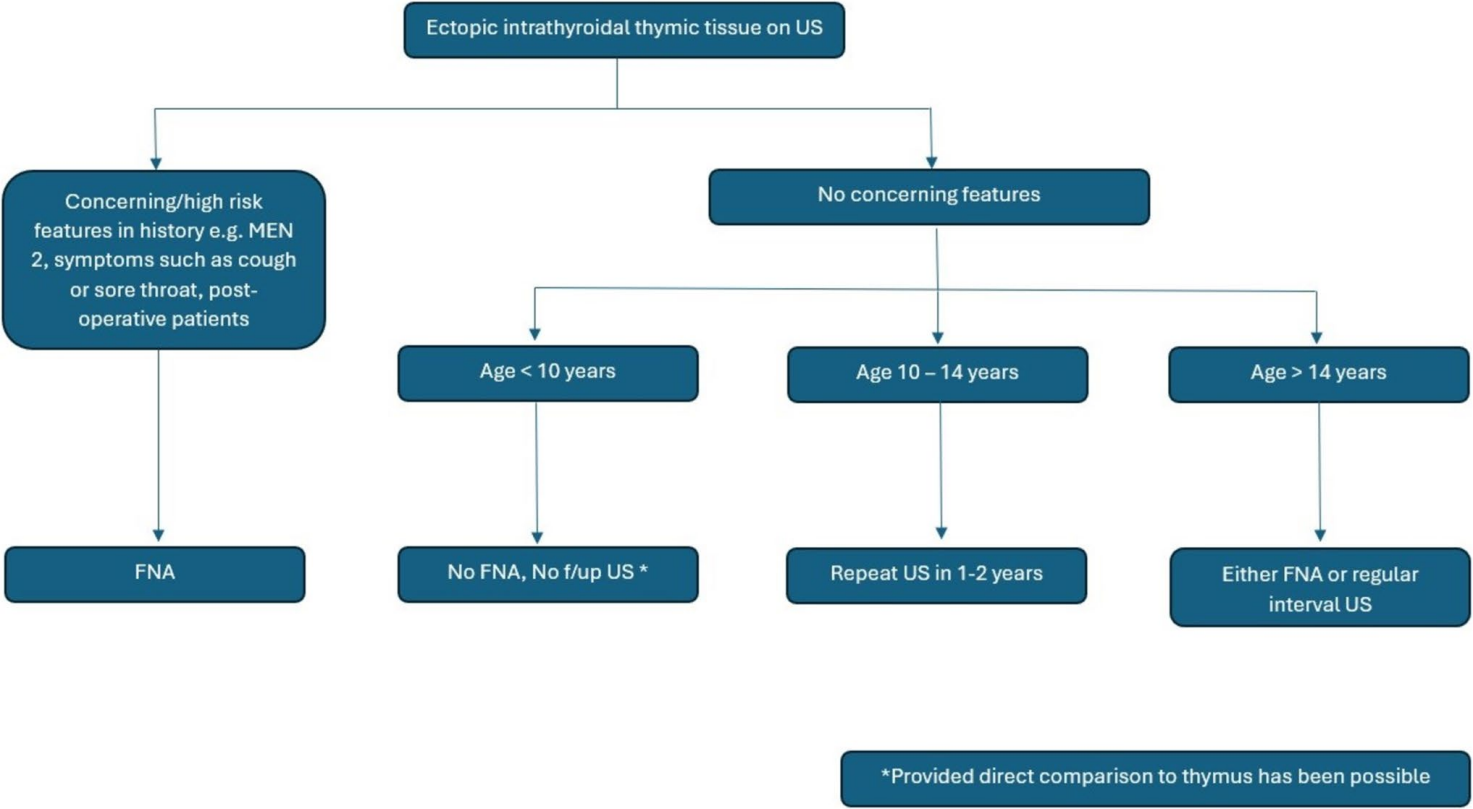
Proposed management algorithm for paediatric patients with ectopic intrathyroidal thymic tissue

We would therefore recommend a separation of children with ectopic intrathyroidal thymic tissue into three groups based on age and the ability to compare directly with normal thymic tissue (Fig. 4). Children under 10 years old with availability of direct comparison to thymus and no relevant personal or family history and no concerning features in the history and clinical examination should not have an FNA biopsy and require no further ultrasound scans. Children between 10 and 14 years old would benefit from a repeat US scan in 1–2 years to assess and compare the stability or involution of the intrathyroidal nodule to the thymus. The third group > 14 years would require either regular interval scans or an FNA biopsy to exclude a thyroid cancer.

A limitation of this study is the low sample size, and a median follow up period of only six months, therefore this is a proposed stratification system which may be considered where there is no diagnostic uncertainty, and in patients without high-risk features. Further validation of this model in larger studies with longer follow-up duration is necessary to draw definitive conclusions regarding the safety of this approach.

In conclusion, this study highlights the need for increased awareness of intrathyroidal thymic tissue among clinicians and the need for a safe standardised diagnostic approach. It provides evidence for the safe use of surveillance scanning in low-risk paediatric patients with thyroid masses that resemble ectopic thymic tissue. Awareness among radiologists of this developmental variant and the value of comparison to normal thymic tissue to aid in its diagnosis has the potential to reduce parental anxiety and general anaesthetic required for FNA associated with tertiary referral for suspected thyroid malignancy. We propose a new classification based on age and visible thymic tissue to aid clinicians and radiologists make a decision on follow up and treatment.

## Data Availability

No datasets were generated or analysed during the current study.
